# CD4^+^CD25^−^Foxp3^+^ T cells play a role in tuberculous hydrothorax rather than malignant hydrothorax

**DOI:** 10.1186/s12967-015-0618-6

**Published:** 2015-08-18

**Authors:** Ying Tang, Li-Ping Peng, Gui-Xiang Qin, Jing-Ting Sun, Li-Jun Xu, Yan-Fang Jiang

**Affiliations:** Department of Respiratory Medicine, The First Hospital, Jilin University, No. 71 Xinmin Street, Changchun, 130021 China; The Center of Tuberculous Meningitis Diagnosis and Treatment, The Infectious Disease Hospital of Changchun, No. 2699 the south line of Changchun to Jilin, Changchun, 130123 China; Key Laboratory for Zoonosis Research, Ministry of Education, The First Hospital, Jilin University, No. 3302 Jilin Road, Changchun, 130032 China

**Keywords:** Tuberculous hydrothorax, Malignant hydrothorax, CD4^+^CD25^+^FoxP3^+^ T cells, CD4^+^CD25^−^FoxP3^+^ T cells

## Abstract

**Background:**

Foxp3^+^ T cells regulate inflammation and tumorigenesis. However, little is known about the role of different subsets of Foxp3^+^ T cells in malignant or tuberculous hydrothorax.

**Methods:**

The numbers of CD4^+^CD25^+^Foxp3^+^, CD4^+^CD25^−^Foxp3^+^ T cells and the levels of some inflammatory cytokines in patients with tuberculous hydrothorax, malignant hydrothorax, and healthy controls (HCs) were examined by flow cytometry and ELISA. The potential association between the numbers of different subsets of Foxp3 + T cells and the values of clinical measures were analyzed.

**Results:**

The numbers of peripheral blood CD4^+^CD25^+^Foxp3^+^ T cells were greater in malignant hydrothorax patients than in HCs, but fewer than those of hydrothorax in patients. The percentages of circulating IL-10^+^ or LAP^+^ CD4^+^CD25^+^Foxp3^+^ T cells were higher than in the hydrothorax in patients with malignant hydrothorax. The numbers of circulating CD4^+^CD25^−^Foxp3^+^ T cells were significantly fewer in patients with tuberculous hydrothorax than in HCs, and both the numbers of circulating CD4^+^CD25^+^Foxp3^+^ and CD4^+^CD25^−^Foxp3^+^ T cells were significantly fewer than in the hydrothorax in patients. Significantly higher percentages of circulating IL-10^+^ or LAP^+^ CD4^+^CD25^+^Foxp3^+^ and CD4^+^CD25^−^Foxp3^+^ T cells were detected in tuberculous hydrothorax patients. The numbers of CD4^+^CD25^+^Foxp3^+^ and CD4^+^CD25^−^Foxp3^+^ T cells were associated with hydrothorax adenosine deaminase (ADA) levels in tuberculous hydrothorax patients, while CD4^+^CD25^+^Foxp3^+^ T cells were associated with carcino-embryonic antigen (CEA) in malignant hydrothorax patients. The concentrations of serum IL-6 and TGF-β in the patients were significantly higher than that in the HCs, but lower than that in the corresponding hydrothorax. A similar pattern of IL-10 was observed in different groups, except that there was no significant difference in the levels of serum IL-10 between the tuberculous hydrothorax patients and HCs.

**Conclusions:**

CD4^+^CD25^−^Foxp3^+^ T cells, which have lower inhibitory function than CD4^+^CD25^+^Foxp3^+^ T cells, may play a role in tuberculous hydrothorax.

## Background

Hydrothorax is a medical condition, which results from the disorder of pleural absorption due to a systemic or local disease. Lung cancer and tuberculosis infection are common factors for the formation of hydrothorax. Previous studies have suggested that immune escape participates in the pathogenesis of malignant hydrothorax or tuberculous hydrothorax [1–3]. It is known that regulatory T cells (Treg) are crucial for peripheral tolerance, and they actively and predominately inhibit both the activation and effector function of autoreactive T cells [4, 5]. However, little is known about the potential role of Tregs in the pathogenesis of malignant hydrothorax and tuberculous hydrothorax.

Treg are characterized by CD4^+^CD25^+^ [6] and express the forkhead family transcription factor (Foxp3), which is necessary for the development and function of Tregs [7–9]. Foxp3 has been described as a highly specific intracellular marker for Tregs. The CD4^+^CD25^+^FoxP3^+^ Tregs have potent suppressive activity to prevent pathogenic damage under inflammatory conditions, and they also have the tendency to keep tumors from host immunologic surveillance [10–12]. Therefore, CD4^+^CD25^+^FoxP3^+^ Tregs can promote immune escape of tumor cells and *Mycobacterium tuberculosis* (*M.tb*). Indeed, high percentages of CD4^+^CD25^+^FoxP3^+^ Tregs have been found in the hydrothorax of patients with tuberculous hydrothorax and malignant hydrothorax [13, 14]. However, a recent study reveals that CD4^+^CD25^−^FoxP3^+^ Tregs develop in the periphery and are derived from CD4^+^CD25^−^ T cells, dependent on environmental antigens presented by dendritic cells (DCs) [15]. Hence, CD4^+^CD25^+^FoxP3^+^ and CD4^+^CD25^−^Foxp3 Tregs are activated by different types of antigens and develop in varying environments [16]. However, the function of CD4^+^CD25^−^Foxp3^+^ Tregs is still unclear. One study indicates that CD4^+^CD25^−^FoxP3^+^ Tregs act similar to that of conventional Tregs to a certain extent [17], but another study reveals that CD4^+^CD25^−^Foxp3^+^ Tregs are different from CD4^+^CD25^+^ Tregs both phenotypically and functionally [18]. IL-6 can convert CD4^+^CD25^+^FoxP3^+^ but not CD4^+^CD25^−^FoxP3^+^ Tregs to Th17 cells [19], and CD4^+^CD25^−^FoxP3^+^ Tregs may retain suppressive function in an inflammatory environment [20]. The difference in their mechanisms of generation and in the sensitivity to IL-6 on their functional properties suggests that they may have different roles in the adaptive immune response [11]. However, there are few researches that distinguish the roles of FoxP3^+^ Tregs between malignant and tuberculous hydrothorax, and there is no study that distinguishes CD4^+^CD25^−^FoxP3^+^ from CD4^+^CD25^+^FoxP3^+^ Tregs during the process of malignant and tuberculous hydrothorax. Given that Tregs express IL-10 and latency associated peptide (LAP), it is unclear how the frequency of IL-10^+^ or LAP^+^ CD4^+^CD25^+^FoxP3^+^ or CD4^+^CD25^−^FoxP3^+^ Tregs changes in patients with malignant and tuberculous hydrothorax.

Tregs can negatively regulate NK cell function, B cell activation, and humoral responses [11, 18]. Previous studies have shown increased numbers of CD4^+^ T cells in both malignant and tuberculous hydrothorax patients [21–23] and decreased numbers of B cells in tuberculous hydrothorax patients [24, 25]. NK cells have potent anti-tumor and anti-tuberculosis activity in malignant and tuberculous hydrothorax [26, 27]. However, whether the frequency of T, B, and NK cells are similar or different between malignant and tuberculous hydrothorax has not been clarified.

In the present study, we tested the hypothesis that CD4^+^CD25^+^Foxp3^+^ and CD4^+^CD25^−^Foxp3^+^ Tregs may play different roles in the pathogenesis of malignant and tuberculous hydrothorax, and we found that both the numbers and secretary function of CD4^+^CD25^+^Foxp3^+^ and CD4^+^CD25^−^Foxp3^+^ T cells changed in the development of tuberculous hydrothorax, while only CD4^+^CD25^+^Foxp3^+^ T cells changed in the malignant hydrothorax.

## Methods

### Subjects

Forty-nine patients with hydrothorax (25 tuberculosis and 24 pulmonary tumors) were recruited sequentially from the inpatient service of the First Hospital of Jilin University from March 2012 to May 2013. Another 23 age-, gender-, and ethnicity-matched healthy volunteers from the Physical Examination Center were recruited as healthy controls (HCs), and they had no historical or current chronic disease and smoking. One patient with tuberculous hydrothorax was diagnosed by the detection of *M.tb* on a hydrothorax smear, and other 24 patients were diagnosed with positive pleural biopsy specimen with granulomatous pleurisy in the absence of evidence of other granulomatous diseases. Patients with malignant hydrothorax were diagnosed by detection of malignant cells in the hydrothorax and/or biopsied pleural specimens. Patients were excluded if she/he received anti-cancer, anti-tuberculosis therapy, corticosteroids, or immunosuppressants for any reason or had renal, hepatic, endocrine diseases, or were pregnant. Written informed consent was obtained from each individual subject, and the experimental protocol was approved by the Ethical Committee of the First Hospital of Jilin University.

### Clinical measurements

The concentrations of serum and hydrothorax carcinoembryonic antigen (CEA) were determined by the chemiluminescence immunoassay using a specific kit, according to the manufacturers’ instruction (Beckman Coulter, Fullerton, USA). The levels of lactate dehydrogenase (LDH) and adenosine deaminase (ADA) in individual samples were determined by the enzyme colorimetry using specific kits (Huachenbio, Shanghai, China) and (Aosibangbio, Yantai, China), respectively, according to the manufacturer’s protocols.

### Cell isolation and flow cytometry

Venous blood (10 mL) and hydrothorax (15 mL) samples were collected and centrifuged at 1,500 rpm for 10 min. The pelleted cells were subjected to density-gradient centrifugation to prepare peripheral blood mononuclear cells and hydrothorax mononuclear cells. The cells (10^6^ cells/tube) were stained in duplicate with fluorescein isothiocyanate (FITC)-anti-CD4 (BD PharMingen, San Diego, USA) and APC-anti-CD25 (BD PharMingen) at room temperature (RT) for 30 min, fixed with 1× Human FoxP3 Buffer A (10 min/RT), and permeabilized with 1× working solution of Human FoxP3 Buffer C for 30 min at RT. After being washed with PBS, the cells were stained with phycoerythrin (PE)-anti-FoxP3 (BD PharMingen) for 30 min and characterized by flow cytometry analysis using a FACS Calibur (BD Biosciences) and FlowJo software (v5.7.2) (TreeStar, Ashland, USA). The cells were gated initially on living lymphocytes, according to the basis of forward and side angle light scattering characteristics. Subsequently, the cells were gated on CD4^+^ T cells to determine the frequency of CD4^+^CD25^+^Foxp3^+^ and CD4^+^CD25^−^Foxp3^+^ Tregs in total CD4^+^ T cells. The numbers of CD4^+^CD25^+^Foxp3^+^ and CD4^+^CD25^−^Foxp3^+^ Tregs in peripheral blood were calculated by the formula (cell numbers = lymphocytes number (gain from routine blood test) × $\frac{{{\text{CD4}}^{ + } }}{\text{lymphocyte}}cells$% × $\frac{{{\text{CD25}}^{{ + {\text{or - }}}} F{\text{oxP3}}^{ + } }}{{{\text{CD4}}^{ + } }}cells$%), and in hydrothorax by the formula (cell numbers = hydrothorax leukocyte numbers (gain from routine hydrothorax test) × mononuclear cells % (gain from routine hydrothorax test) × $\frac{{{\text{CD4}}^{ + } }}{\text{lymphocyte}}cells$% × $\frac{{{\text{CD25}}^{{ + {\text{or - }}}} F{\text{oxP3}}^{ + } }}{{{\text{CD4}}^{ + } }}cells$%).

To determine the other types of lymphocytes, the prepared peripheral blood mononuclear cells and hydrothorax mononuclear cells were stained with FITC-anti-CD4, PE-anti-CD8, peridinin chlorophyll-a protein (PerCP)-anti-CD3 (BD Tritest, San Jose, CA, USA), or FITC-anti-CD5, PE-anti-CD19 (BD Simultest, San Jose, CA, USA), or FITC-anti-CD3, PE-anti-CD16CD56 (BD Simultest), respectively at RT for 30 min. After being washed, the cells were characterized by flow cytometry analysis. The cells were gated initially on living lymphocytes. The numbers of CD3^+^, CD3^+^CD4^+^, CD3^+^CD8^+^ T cells, CD5^+^CD19^+^ B cells, and CD3^−^CD16CD56^+^ NK cells in peripheral blood were calculated according to the formula (cell numbers = lymphocyte absolute numbers (gain from routine blood test) × $\frac{{{\text{CD3}}^{ + } or{\text{ CD3}}^{ + } {\text{CD4}}^{ + } or{\text{ CD3}}^{ + } {\text{CD8}}^{ + } \, or{\text{ CD5}}^{ + } {\text{CD19}}^{ + } \, or{\text{ CD3}}^{ - } {\text{CD16CD56}}^{ + } \, }}{\text{lymphocyte}}cells$%), and in hydrothorax by the formula (cell numbers = hydrothorax leukocyte number (gain from routine hydrothorax test) × mononuclear cells % (gain from routine hydrothorax test) × $\frac{{{\text{CD3}}^{ + } or{\text{ CD3}}^{ + } {\text{CD4}}^{ + } or{\text{ CD3}}^{ + } {\text{CD8}}^{ + } \, or{\text{ CD5}}^{ + } {\text{CD19}}^{ + } \, or{\text{ CD3}}^{ - } {\text{CD16CD56}}^{ + } \, }}{\text{lymphocyte}}cells$%).

### The detection of IL-10^+^ or LAP^+^ CD4^+^Foxp3^+^ Tregs

The peripheral blood mononuclear cells and hydrothorax mononuclear cells (10^6^ cells/well) were stimulated in duplicate with 50 ng/mL of phorbol myristate acetate (PMA) and 1.0 μg/mL of ionomycin (Sigma-Aldrich, St. Louis, USA) in complete RPMI 1640 medium for 2 h at 37°C in 5% CO_2_ and exposed to Brefeldin A (GolgiPlug; BD Biosciences) for 4 h. The cells cultured in medium alone without stimulation served as negative controls. After being washed, the cells were stained in duplicate with PerCP-anti-CD4 and APC-anti-CD25, fixed, permeabilized, followed by intracellular staining with PE-anti-FoxP3 and Alexa Fluor 488-anti-IL-10 (eBioscience, San Diego, USA) or control IgG1 (eBioscience, San Diego, USA) for 30 min, or stained with PerCP-anti-CD4, APC-anti-CD25, Alexa Fluor 488-anti-LAP (eBioscience) or control IgG1 (eBioscience, San Diego, USA), fixed, permeabilized, and followed by PE-anti-FoxP3 for 30 min. Finally, the cells were characterized by flow cytometry analysis. The cells were gated initially on living lymphocytes, and then on CD4^+^ T cells. Subsequently, the CD4^+^CD25^+^Foxp3^+^ and CD4^+^CD25^−^Foxp3^+^ Tregs were analyzed. The numbers of IL-10^+^ or LAP^+^ CD4^+^Foxp3^+^ Tregs were calculated by the formula (cell numbers = CD4^+^CD25^+*or*−^Foxp3^+^ numbers × $\frac{{{\text{IL - 10}}^{ + } {\text{or LAP}}^{ + } }}{{{\text{CD4}}^{ + } {\text{CD25}}^{{ + {\text{or - }}}} {\text{Foxp3}}^{ + } }}cells$%). At least 20,000 events were analyzed for each sample in all flow cytometry analysis.

### Measurement of cytokines

The concentrations of IL-6 and IL-10 were determined by cytometric bead array (CBA) using a commercial kit (BD Biosciences, San Joes, USA), and were quantified using the Cell Quest Pro and CBA software (Becton–Dickinson) on a FACS Calibur cytometry. The concentrations of transforming growth factor-β (TGF-β) in individual subjects were determined by enzyme linked immunosorbent assay (ELISA) using a human TGF-β ELISA kit (R&D Systems, Minneapolis, USA) and were calculated, according to the standard curve established using the recombinant TGF-β provided. The limitation of detection for IL-6, IL-10 or TGF-β was 2.5, 3.3, or 15.4 pg/mL, respectively.

### Statistical analyses

Data are expressed as the median and range unless specified. The difference among the three groups was analyzed by ANOVA and post hoc Bonferroni analysis, and between two groups was analyzed by the Wilcoxon rank sum test using SPSS 16.0 software. A two-sided P value <0.05 was considered statistically significant.

## Results

### Demographic and clinical characteristics of patients

As shown in Table 1, there was no significant difference in the distribution of age, gender, and the numbers of white blood count (WBC) among three groups, also in the numbers of lymphocytes between the peripheral blood from malignant hydrothorax patients and HCs, while the numbers of lymphocytes in the peripheral blood from tuberculous hydrothorax patients were significantly less than those in the malignant hydrothorax patients and HCs (p < 0.05). In addition, the numbers of WBC, mononuclear cells and ADA in the malignant hydrothorax were significantly less than that in the tuberculous hydrothorax (p < 0.05), but the levels of CEA and LDH in the malignant hydrothorax were higher than that in the tuberculous hydrothorax (p < 0.05).

**Table 1:** The demographic and clinical characteristics of subjects

Characteristics	Tuberculous hydrothorax (n = 25)	Malignant hydrothorax (n = 24)	HCs (n = 23)
Male/female	13/12	12/12	12/11
Age	45 (23–67)	55 (39–67)	48 (28–68)
Blood WBC (×10^9^/L)	6.15 ± 1.94	6.59 ± 1.62	6.16 ± 1.33
Blood Lymphocyte (×10^9^/L)	1.19 ± 0.38*	1.58 ± 0.33^#^	1.50 ± 0.35
Pleural fluid			
WBC (×10^6^/mL)	2,340 (650–5,120)	1,730 (680–2,670)^#^	_
Mononuclear cells (×10^6^/mL)	1,808 (520–4,096)	1,408 (544–2,286)^#^	_
CEA (ng/mL)	1.69 (0.46–5.8)	87.6 (1.43–951.5)^#^	_
LDH (U/L)	375 (192–1,249)	726 (208–1,180)^#^	_
ADA (U/L)	52.6 (3.5–113.2)	23.2 (3.7–48.7)^#^	_

*WBC* white blood cell, *CEA* carcino-embryonic antigen, *LDH* lactate dehydrogenase, *ADA* adenosine deaminase, – not available.

* P < 0.05 vs. the HCs; ^#^P < 0.05 vs. the tuberculous hydrothorax patients; the normal values for CEA, LDH, and ADA are 0–5 ng/mL, 135–226 U/L, and 0–25 U/L, respectively.

### The numbers of T, B, and NK cells in tuberculous and malignant hydrothorax patients

**Fig. 1:**
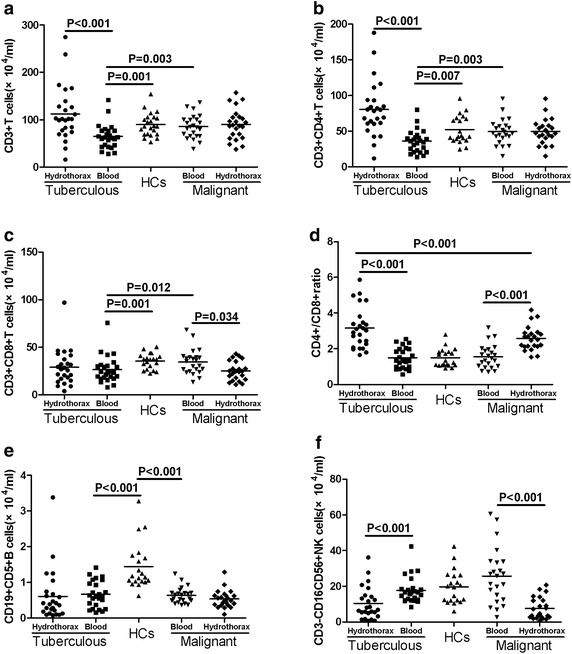
The numbers of different subsets of lymphocyte in tuberculous and malignant hydrothorax patients. The peripheral blood and hydrothorax mononuclear cells were stained with specific antibodies respectively. The numbers of blood and hydrothorax CD3^+^, CD3^+^CD4^+^ T, CD3^+^CD8^+^ T, CD19^+^CD5^+^ B cells, and CD3^−^CD16/56^+^ NK cells were characterized by flow cytometry, and the ratios of CD4^+^ to CD8^+^ T cells were calculated in individual groups of subjects. Data shown are the mean values of individual subjects. **a**–**c** The numbers of CD3^+^, CD3^+^CD4^+^, and CD3^+^CD8^+^ T cells.** d** The ratios of CD4^+^ to CD8^+^ T cells. **e**, **f** The numbers of CD19^+^CD5^+^ B cells and CD3^−^CD16/56^+^ NK cells. The *horizontal lines* show the median values of individual groups. *HCs* healthy controls.

Given that T, B, and NK cells play varying function in the pathogenesis of tuberculous and malignant hydrothorax, we first characterized the numbers of T, B and NK cells in peripheral blood and hydrothorax samples from tuberculous and malignant hydrothorax patients by flow cytometry analysis. The numbers of circulating CD3^+^, CD3^+^CD4^+^, and CD3^+^CD8^+^ T cells in the tuberculous hydrothorax patients were significantly less than that in the malignant hydrothorax patients (p < 0.05 for all) and HCs (p < 0.01 for all) (Fig. 1a–c). The numbers of circulating CD19^+^CD5^+^ B cells were similar between the tuberculous hydrothorax and malignant hydrothorax patients and were significantly less than those in the HCs (p < 0.001, Fig. 1e). Further analysis indicated that the numbers of CD3^+^ and CD3^+^CD4^+^ T cells in the tuberculous hydrothorax were significantly greater than that in blood (p < 0.001, Fig. 1a, b). However, there was no significant difference in the numbers of CD3^+^CD8^+^ T cells between blood and hydrothorax from tuberculous hydrothorax patients (Fig. 1c). As a result, the ratios of hydrothorax CD4^+^ to CD8^+^ T cells were greater than that in the blood of the tuberculous hydrothorax patients (p < 0.001, Fig. 1d). In contrast, the numbers of hydrothorax CD3^−^CD16/CD56^+^ NK cells were significantly less than that in blood in both the tuberculous and malignant hydrothorax patients (p < 0.001, Fig. 1f).

### Characterization of CD4^+^CD25^+^Foxp3^+^ and CD4^+^CD25^−^Foxp3^+^ Treg in patients with tuberculous hydrothorax and malignant hydrothorax

**Fig. 2:**
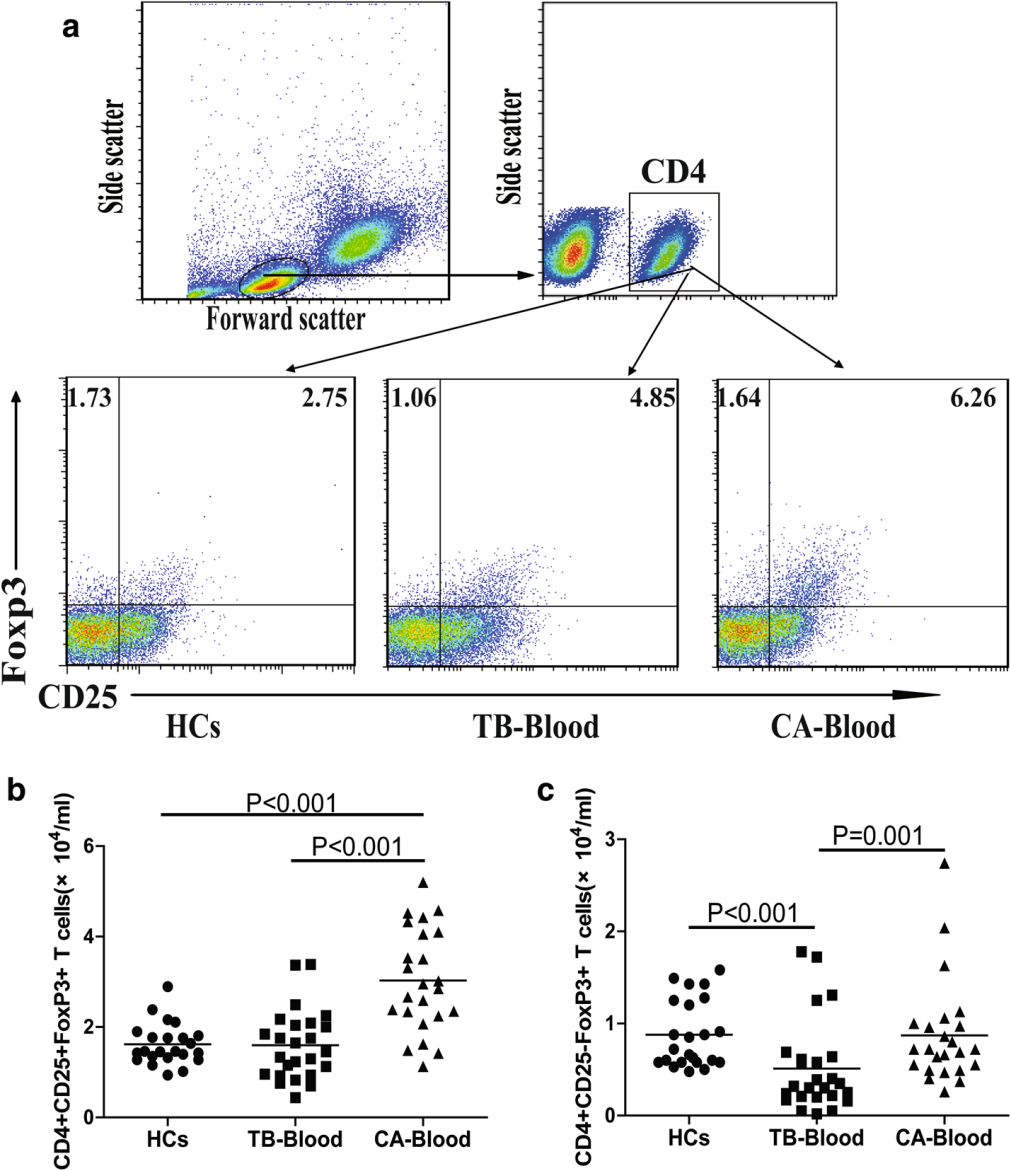
Flow cytometry analysis of the numbers of different phenotypes of peripheral blood Foxp3^+^ T cells. The peripheral blood and hydrothorax mononuclear cells were stained with anti-CD4 and anti-CD25, fixed and permeabilized, followed by staining with anti-Foxp3. The cells were first gated on living lymphocytes and then on CD4^+^ cells, and the numbers of blood CD4^+^CD25^+^Foxp3^+^ and CD4^+^CD25^−^Foxp3^+^ T cells were characterized by flow cytometry analysis. Data shown are representative charts and expressed as the means of individual subjects. **a** Representative charts; **b** the numbers of CD4^+^CD25^+^Foxp3^+^ T cells; **c** the numbers of CD4^+^CD25^−^Foxp3^+^ T cells. The *horizontal lines* show the median values of individual groups. *TB* tuberculous patients, *CA* malignant patients.

**Fig. 3:**
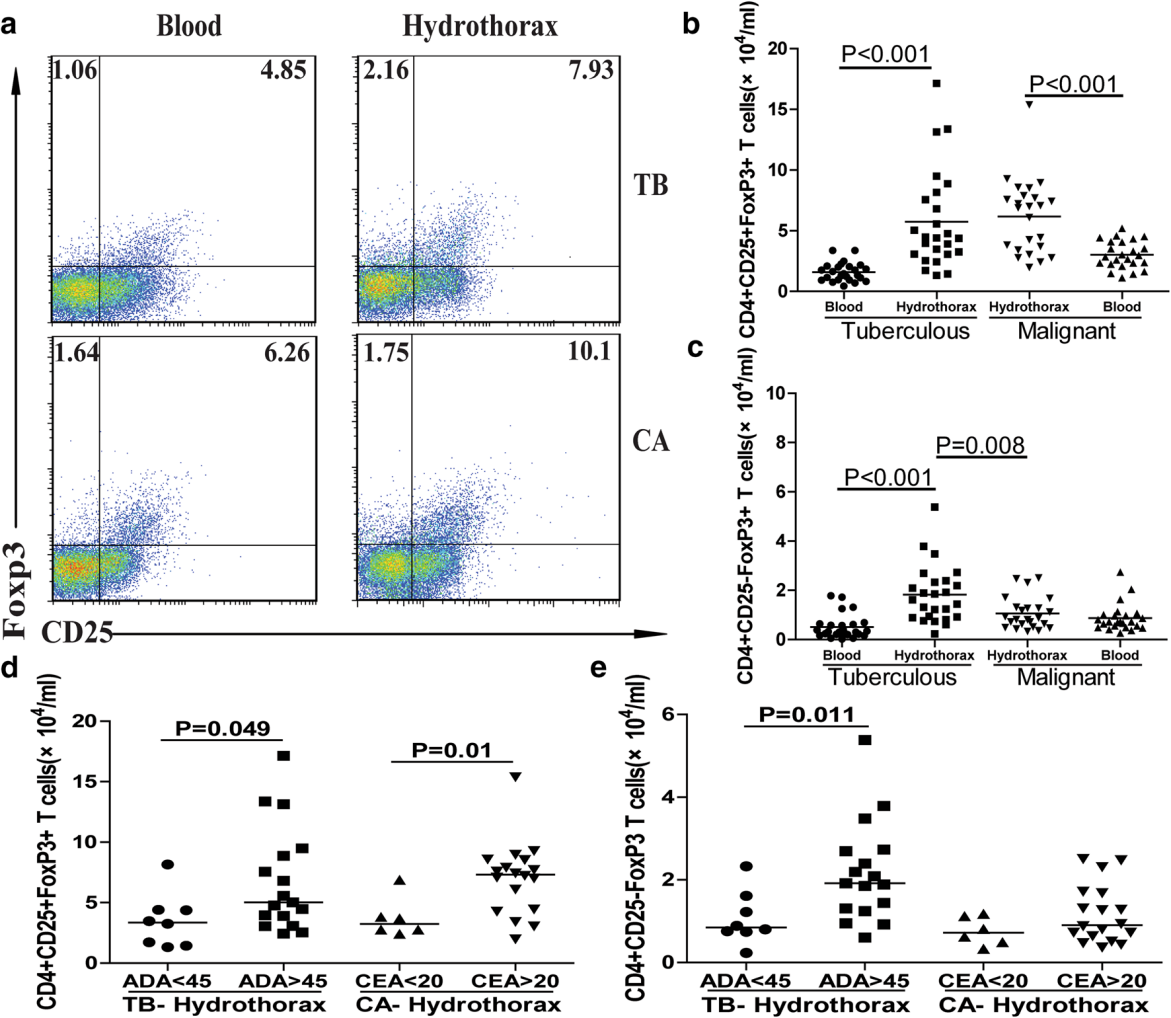
Flow cytometry analysis of the numbers of hydrothorax Foxp3^+^ T cells in tuberculous and malignant hydrothorax patients. The peripheral blood and hydrothorax mononuclear cells were stained with anti-CD4 and anti-CD25, fixed and permeabilized, followed by staining with anti-Foxp3. The cells were first gated on living lymphocytes and on CD4^+^ cells, and the numbers of blood and hydrothorax CD4^+^CD25^+^Foxp3^+^ and CD4^+^CD25^−^Foxp3^+^ T cells were characterized by flow cytometry analysis. Subsequently, the numbers of hydrothorax CD4^+^CD25^+^Foxp3^+^ and CD4^+^CD25^−^Foxp3^+^ T cells were stratified, according to the levels of hydrothorax ADA = 45 in tuberculous hydrothorax patients and CEA = 20 in malignant hydrothorax patients. Data shown are representative charts and expressed as the means of individual subjects. **a** Representative charts; **b** the numbers of CD4^+^CD25^+^Foxp3^+^ T cells; **c** the numbers of CD4^+^CD25^−^Foxp3^+^ T cells. **d** stratification analysis of the numbers of CD4^+^CD25^+^Foxp3^+^ T cells. **e** Stratification analysis of the numbers of CD4^+^CD25^−^Foxp3^+^ T cells. The *horizontal lines* show the median values of individual groups.

Next, we characterized the numbers of Tregs. The numbers of circulating CD4^+^CD25^+^Foxp3^+^ Tregs in the malignant hydrothorax patients were significantly greater than those in the tuberculous hydrothorax patients and HCs (P < 0.001 for both, Fig. 2b), while the numbers of circulating CD4^+^CD25^−^Foxp3^+^ Tregs in the tuberculous hydrothorax patients were significantly less than that in the HCs and malignant hydrothorax patients (P < 0.05 for both, Fig. 2c). Further analysis indicated that the numbers of CD4^+^CD25^+^Foxp3^+^ Tregs were similar between the tuberculous and malignant hydrothorax and were significantly greater than those in the blood of both groups of patients (p < 0.001 for both, Fig. 3a, b). In addition, the numbers of CD4^+^CD25^−^Foxp3^+^ Tregs in the tuberculous hydrothorax were significantly greater than those in the blood (p < 0.001) and malignant hydrothorax (p = 0.008, Fig. 3c).

### The relationship between the numbers of CD4^+^CD25^+^Foxp3^+^ or CD4^+^CD25^−^Foxp3^+^ Tregs and clinical parameters

Because ADA and CEA are important biomarkers for tuberculous hydrothorax and malignant hydrothorax, respectively. We further stratified patients and analyzed the potential association between the numbers of Tregs and the levels of hydrothorax ADA or CEA in tuberculous hydrothorax or malignant hydrothorax patients. Considering that the level of ADA >45 U/L is usually recognized to be characteristic of tuberculous hydrothorax, while CEA >20 U/L is characteristic of malignant hydrothorax, we used ADA = 45 U/L and CEA = 20 U/L as boundaries for stratification. The numbers of CD4^+^CD25^+^Foxp3^+^ and CD4^+^CD25^−^Foxp3^+^ Tregs in the tuberculous hydrothorax with ADA >45 U/L were significantly greater than those in the patients with ADA <45 U/L (P = 0.049, P = 0.011, Fig. 3d, e). Similarly, the numbers of CD4^+^CD25^+^Foxp3^+^, but not CD4^+^CD25^−^Foxp3^+^, Tregs in the malignant hydrothorax with CEA >20 U/L were significantly greater than those in the patients with CEA <20 U/L (P = 0.01, Fig. 3d).

### The expression of IL-10 or LAP on CD4^+^CD25^+^Foxp3^+^ and CD4^+^CD25^−^Foxp3^+^ T cells in malignant and tuberculous hydrothorax patients

**Fig. 4:**
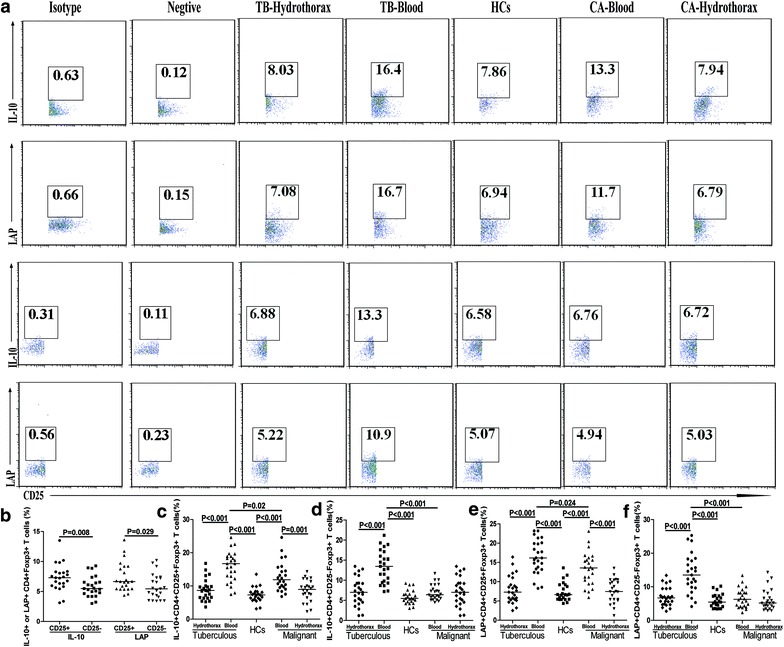
Flow cytometry analysis of the percentages of peripheral blood and hydrothorax IL-10^+^ or LAP^+^ CD4^+^CD25^+^Foxp3^+^ and CD4^+^CD25^−^Foxp3^+^ T cells. The peripheral blood and hydrothorax mononuclear cells were stained with anti-CD4 and anti-CD25, fixed and permeabilized, followed by staining with anti-Foxp3 and anti-IL-10 (or control IgG1) or stained with anti-CD4, anti-CD25, and anti-LAP (or control IgG1), fixed and permeabilized, followed by staining with anti-Foxp3, respectively. The blood or hydrothorax mononuclear cells cultured in medium alone served as negative controls. The cells were first gated on CD4^+^ and then on CD4^+^CD25^+^Foxp3^+^ or CD4^+^CD25^−^Foxp3^+^ cells, followed by determining the percentages of IL-10^+^ or LAP^+^ CD4^+^CD25^+^Foxp3^+^ and CD4^+^CD25^−^Foxp3^+^ T cells. Data shown are representative charts and expressed as the means of individual subjects. **a** Representative charts. **b** The percentages of different phenotypes of Foxp3^+^ cells in the HCs. **c**–**f** The percentages of different phenotypes of Foxp3^+^ T cells in individual groups of subjects. The *horizontal lines* show the medians of individual groups.

IL-10 and LAP are expressed by CD4^+^CD25^+^Foxp3^+^ and CD4^+^CD25^−^Foxp3^+^ Tregs and are important for their function. Accordingly, we characterized IL-10^+^ or LAP^+^ Tregs by flow cytometry. We found the frequency of IL-10^+^ or LAP^+^ CD4^+^CD25^+^Foxp3^+^ Treg was significantly higher than that of IL-10^+^ or LAP^+^ CD4^+^CD25^−^Foxp3^+^ Tregs in the HCs (p < 0.05, Fig. 4b). The percentages of circulating IL-10^+^CD4^+^CD25^+^Foxp3^+^ Tregs in the tuberculous hydrothorax patients were significantly higher than that of hydrothorax (p < 0.001) and that in the blood of malignant hydrothorax patients (p = 0.02), which was also significantly higher than that in the hydrothorax (p = 0.001) and in the HCs (p < 0.001, Fig. 4c). A similar pattern of LAP^+^CD4^+^CD25^+^Foxp3^+^ T cells was observed in the different groups of subjects (Fig. 4e). Furthermore, the percentages of circulating IL-10^+^CD4^+^CD25^−^Foxp3^+^ and LAP^+^CD4^+^CD25^−^Foxp3^+^ T cells in the tuberculous hydrothorax patients were significantly higher than that of hydrothorax and that in the HCs and malignant hydrothorax patients (Fig. 4d, f).

### The levels of different cytokines in tuberculous and malignant hydrothorax patients

**Fig. 5:**
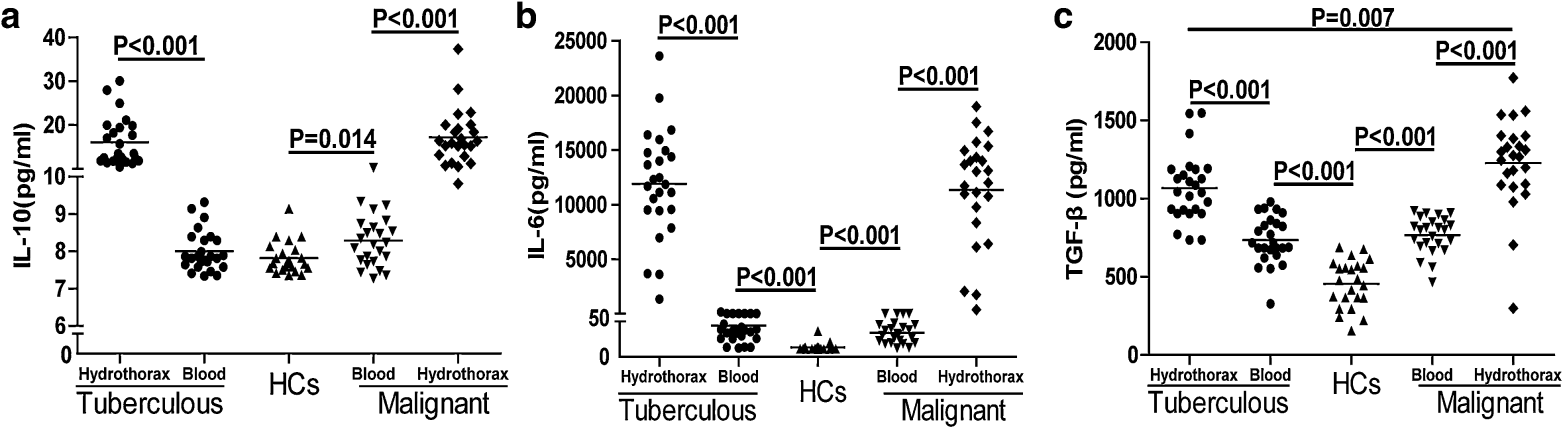
The concentrations of peripheral blood and hydrothorax cytokines in different groups of subjects. The concentrations of peripheral blood and hydrothorax indicated cytokines in individual subjects were determined by ELISA and CBA. **a**–**c** Data shown are the means of cytokines in individual subjects, and the *horizontal lines* indicate the median values of individual groups.

The concentrations of serum IL-6 and TGF-β in both the tuberculous and malignant patients were significantly higher than that in the HCs, while lower than that in the hydrothorax from corresponding patients (p < 0.05, Fig. 5b, c), A similar pattern of IL-10 was observed in the different groups of samples, except that there was no significant difference in the levels of serum IL-10 between the tuberculous hydrothorax patients and HCs (Fig. 5a). Furthermore, the levels of TGF-β in the malignant hydrothorax were significantly higher than that in the tuberculous hydrothorax (p < 0.05).

## Discussion

Tuberculous and malignant hydrothorax represent benign and malignant types of pleural effusion, respectively. Previous studies have indicated that Foxp3^+^ Tregs regulate immune responses in the development of tuberculosis and the growth of malignant tumors [28, 29]. In the present study, we found the numbers of peripheral blood CD4^+^CD25^+^Foxp3^+^ T cells were greater in malignant hydrothorax patients than HCs, while less than the malignant hydrothorax. In addition, we found increased levels of IL-10 and TGF-β, which can negatively regulate effector T cells [30]. Tregs can accumulate in the early stage of tumorigenesis [31] and play a suppressive role through cell-to-cell contact mechanisms [32]. The increased Tregs may promote cancer progression by interfering with immune surveillance because Tregs are an important inhibitory factor in the anti-tumor immunity. Hence, therapeutic targeting of Tregs may be promising for the inhibition of tumor growth.

Tregs also participate in the immune responses against *M.tb*. Our data about the numbers of blood CD4^+^CD25^+^Foxp3^+^ T cells in tuberculous patients is different from the study by Semple and colleagues [33], which may be because the numbers of Tregs are reported by frequency in their research while absolute numbers in ours, and the decreased numbers of lymphocyte numbers in peripheral blood may contribute to this difference. Nowadays, CD4^+^CD25^−^FoxP3^+^ Tregs have been recognized as another subset of CD4^+^FoxP3^+^ Tregs. In the present study, we found that the numbers of circulating CD4^+^CD25^−^FoxP3^+^ Tregs in the tuberculous hydrothorax patients were significantly less than those in the malignant patients and HCs, however, the numbers of CD4^+^CD25^−^FoxP3^+^ Tregs in the tuberculous hydrothorax were significantly greater. The greater numbers of CD4^+^CD25^−^FoxP3^+^ Tregs in the tuberculous hydrothorax may stem from redistribution of this population of T cells from peripheral blood to the inflammatory site during the process of *M.tb* infection [13]. Alternatively, CD4^+^CD25^−^Foxp3^+^ Tregs may constitute a peripheral reservoir of CD4^+^CD25^+^Foxp3^+^ Tregs [34].

Further stratification analysis indicated that the increased numbers of CD4^+^CD25^+^FoxP3^+^ and CD4^+^CD25^−^FoxP3^+^ Treg in the tuberculous hydrothorax were associated with the levels of ADA in tuberculous patients. Given that the ADA can reflect T cell activation, the increased CD4^+^ FoxP3^+^ T cells may represent the extent of the inflammatory reaction. Moreover, we found significantly greater numbers of CD4^+^CD25^+^FoxP3^+^ T cells in the malignant hydrothorax patients with higher levels of hydrothorax CEA. The level of CEA is a valuable prognostic factor in patients with lung cancer, and the levels of serum CEA are correlated negatively with the survival periods of patients with lung cancer [7]. The association of greater numbers of malignant hydrothorax CD4^+^CD25^+^FoxP3^+^ T cells with the higher levels of CEA suggests that CD4^+^CD25^+^FoxP3^+^ T cells may positively promote the growth of tumors.

IL-10 and TGF-β are the main cytokines which Tregs secreted, however, few researches have been designed to distinguish the function of CD4^+^CD25^+^FoxP3^+^ from CD4^+^CD25^−^FoxP3^+^ T cells. In the present study we found a significantly lower frequency of IL-10^+^ and LAP^+^ CD4^+^CD25^−^Foxp3^+^ Tregs compared to IL-10^+^ and LAP^+^ CD4^+^CD25^+^Foxp3^+^ Tregs in HCs. These data suggest that some CD4^+^CD25^−^Foxp3^+^ Tregs may have no inhibitory function. Considering that early activated and uncommitted effector T cells, such as Th17, can express Foxp3, it is possible that some CD4^+^CD25^−^Foxp3^+^ Tregs may be early activated and uncommitted T cells [16, 35].

In addition, we found the percentages of circulating IL-10^+^ and LAP^+^ CD4^+^CD25^+^FoxP3^+^ Tregs in the tuberculous and malignant hydrothorax patients, especially in tuberculous patients, were higher than that in the HCs, and also higher than the corresponding hydrothorax. The lower frequency of IL-10^+^ and LAP^+^ CD4^+^CD25^+^FoxP3^+^Treg in the hydrothorax may be attributed to the inhibition of inflammatory cytokines, such as IL-6 [36]. Indeed, we detected significantly higher levels of IL-6 in both the tuberculous hydrothorax and malignant hydrothorax. In addition, the changes in the frequency of IL-10^+^ and LAP^+^ CD4^+^CD25^−^FoxP3^+^ Tregs were only found in tuberculous hydrothorax patients, which suggested that CD4^+^CD25^−^FoxP3^+^ Tregs might not be involved in the pathogenic process of tumorigenesis. Although there were significantly greater numbers of CD4^+^CD25^+^FoxP3^+^ Tregs in the peripheral blood of malignant hydrothorax patients than those in the tuberculous patients, the frequency of IL-10^+^ or LAP^+^ CD4^+^CD25^+^FoxP3^+^ Tregs was significantly lower. These data suggest the *M.tb* infection may enhance the immunity suppression of Tregs, which need to be further studied.

Moreover, we detected lower levels of TGF-β in the tuberculous hydrothorax than that in the malignant hydrothorax, as well as fewer numbers of CD3^+^, CD3^+^CD4^+^, and CD3^+^CD8^+^ T cells in the peripheral blood of tuberculous hydrothorax than that in the malignant hydrothorax patients. The different levels of such cytokines and cells may serve as biomarkers for distinguishing tuberculous hydrothorax from malignant hydrothorax.

Our findings suggest that CD4^+^CD25^−^Foxp3^+^ T cells may have lower inhibitory function than CD4^+^CD25^+^Foxp3^+^ T cells. CD4^+^CD25^−^Foxp3^+^ T cells may play a role in tuberculous rather than malignant hydrothorax. CD4^+^CD25^+^Foxp3^+^ Tregs may participate in the inflammation process of *M.tb* infection and promote the growth of tumors. We are interested in further studying the dynamic changes of different subsets of Foxp3^+^ Tregs and the molecular mechanisms underlying their actions.

## Abbreviations

HCs: healthy controls
CBA: cytometric bead array
ELISA: enzyme-linked immunosorbent assay
LDH: lactate dehydrogenase
ADA: adenosine deaminase
CEA: carcino-embryonic antigen
Treg: regulatory T cells
Foxp3: the forkhead family transcription factor
*M.tb*: *Mycobacterium tuberculosis*
DCs: dendritic cells
LAP: latency associated peptide
FITC: fluorescein isothiocyanate
RT: room temperature
PE: phycoerythrin
PerCP: peridinin chlorophyll-*a* protein
PMA: phorbol myristate acetate
TGF-β: transforming growth factor-β
WBC: white blood count
